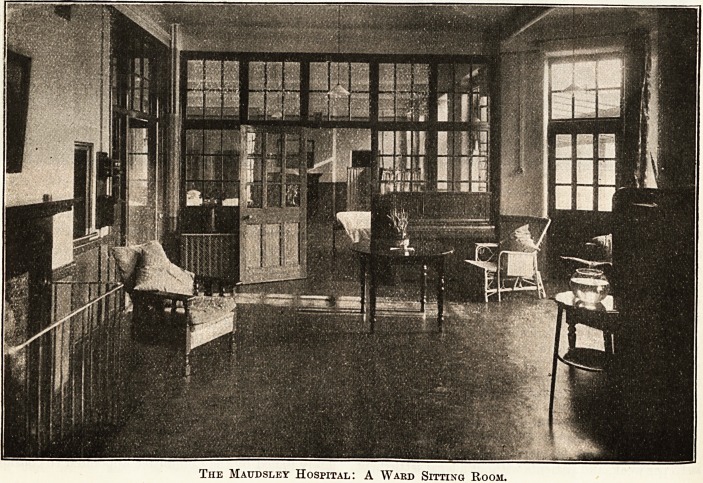# Opening of Maudsley Hospital—A New Departure in the Treatment of Insanity

**Published:** 1923-03

**Authors:** 


### OPENING OF MAUDSLEY HOSPITAL.

### A NEW DEPARTURE IN THE TREATMENT OF INSANITY.

THE opening of the Maudsley Hospital, Denmark Hill, by the Minister of Health, Sir A. Griffith-Boscawen, is an event of the utmost importance in the history of mental institutions in this country. As our readers are aware, the hospital was founded and endowed by the late Dr. Henry Maudsley, and given by him to the London County Council, which becomes the first public authority in England to provide for the voluntary treatment of early types of mental disorder apart from certified cases. Hitherto those suffering from an early and recoverable type of mental trouble, if not possessed of ample means, have had no chance of treatment unless they became certifiable and were admitted into an asylum. At the Maudsley Hospital all patients are voluntary, an in-patient can leave at his own wish on twenty-four hours' notice, and an undertaking is given that no case will be certified in the hospital either for retention there or for transfer to a mental hospital elsewhere.

THE MAUDSLEY HOSPITAL: THE EXTERIOR.

### THE ATMOSPHERE OF HOME AND HOPE.

Maudsley Hospital, which has been erected under the supervision of Mr. W. C. Clifford Smith, Mental Hospitals' Engineer to the London County Council, is a plain building of red brick, and stands facing King's College Hospital. The situation is exceedingly convenient, and the matron assured me that every one would soon grow accustomed to the noise of the trams which run directly past the hospital. Inside everything has been done to escape from the dullness and formality unhappily too often associated with institutions. There is plenty of air, plenty of light, and plenty of colour. It is extraordinarily pleasant to find so much of the real spirit of home in the ward sitting-rooms, with their fresh curtains, their large, comfortable arm-chairs with bright cushions of blue and purple, their well-chosen pictures. On the dining tables are flowers floating in blue bowls, while here and there are growing bulbs ; there are pianos and bookshelves with really attractive books. About the hospital is an air of spacious comfort and an indefinable atmosphere of hope. It would surely be easy to get well here where there is nothing to distress the mind or to weary the eye and where order reigns allied with beauty. Outside are lawns and flower-beds, and a couple of tennis-courts and a Badminton court are being made. In-patients will not necessarily be confined to the house and grounds—they will be allowed out of hospital to such extent as is considered beneficial by Dr. Mapother, the Medical Superintendent.

### HOSPITAL ACCOMMODATION.

Accommodation is available in the hospital for 157 patients, and there is, of course, an out-patients' department. I was shown over six wards, all bright and airy, each ward containing 24 beds, 72 of these being for men and 72 for women. There are also 13 charming little private rooms for female patients, who have a delightful common sitting-room on the ground floor and a special dining-room and garden. The charge in these rooms is six to seven guineas a week. The wards have verandahs which will accommodate several beds that patients may sleep out of doors. Each ward unit is self-contained, with a small consulting-room for the doctor, and a large room full of numbered lockers for the patients' clothes. There are also a few separate bedrooms for those for whom it may be particularly desirable that they should be alone.

### A COMPREHENSIVE SCHEME.

Though intended primarily for early treatment of mental disease, the hospital is also designed to afford opportunities for diagnosis of cases in which special facilities are necessary, for observation of those cases possessing unusual scientific interest, for research, and for the study of psychological medicine. Arrangements will be made for clinical instruction and for lectures, and it is hoped that the hospital will be one of the recognised schools of the University of London. The medical staff consists of the Medical Superintendent and four whole-time Medical Officers, with the voluntary help of a considerable number of qualified medical men and women as clinical assistants. The services of consultant specialists will be available for patients in regard to surgical, gynæcological and obstetric conditions and disorders of eye, ear, nose and throat. A dental surgeon will also attend. Both in-patients and out-patients will be provided with the means of treatment necessary for their special disabilities—drugs and organic extracts, various modes of pyscho-therapy, hydro-therapy, electricity, massage and remedial exercises. Something of a new departure in the treatment of excitable patients is the use of continuous hot baths instead of drugs—patients being kept in them for some days at a time.

THE NURSES' HOME.

On leaving the hospital the matron kindly took me into the Nurses' Home, which is a roomy, old-fashioned house, admirably furnished and equipped. The actual nursing strength is to consist of a matron and assistant matron, seven sisters, eighteen staff nurses and twenty-three probationers. There are also a few male nurses. The Nurses' Home has room only for the assistant matron and nineteen nurses, and the majority of the nursing staff is, therefore, non-resident. The sisters and staff nurses are all in possession of full general training and the standard of efficiency is high. An eight-hour working day is in force, and members of the staff on day duty will have one day a week off, and on night duty two nights a week off.

COST AND ELIGIBILITY.

The present cost of maintenance of an in-patient is £5 per week, and patients without a legal settlement in the County of London cannot be received unless they are prepared to pay this sum. Those with a legal settlement, however, will simply be required to contribute as much as they can properly afford. It is specially desired to co-operate in every way with members of the medical profession outside the hospital, and it will be a general rule to refer to the patient's regular medical attendant. Doctors who care to bring out-patients and to inquire personally about in-patients recommended by them will be welcomed, and if they cannot make personal inquiry a brief report will be sent if requested. No one will be received as an in-patient without the approval of the Medical Superintendent or his deputy, and this will generally involve a personal interview.

THE VERANDAH FOR OPEN-AIR TREATMENT.

IMPORTANCE OF EARLY TREATMENT.

The establishment of the Maudsley Hospital, as the Minister of Health pointed out at its opening, marks a new era in mental treatment. He hoped that before long Parliament would deal with the early treatment of recoverable cases and grant the necessary powers to carry out elsewhere the great experiment that day started. He was glad to observe that special attention had been paid to the selection of the nursing staff. Many unjust aspersions had of late been cast on mental nurses, but he believed that a general hospital training was the best to fit them for work in such a hospital as that. In the report lately issued by the Committee appointed to consider the administration of mental hospitals, the provision of early treatment and the concentration of research were recommended, together with a higher proportion of hospital trained nurses, and the availability of consultants. All these matters had been foreseen in that institution. They were, so to speak, anticipating what Parliament might accomplish. The hospital was a new departure and a public recognition of a new principle.

THE MAUDSLEY HOSPITAL: A WARD SITTING ROOM.

## Figures and Tables

**Figure f1:**
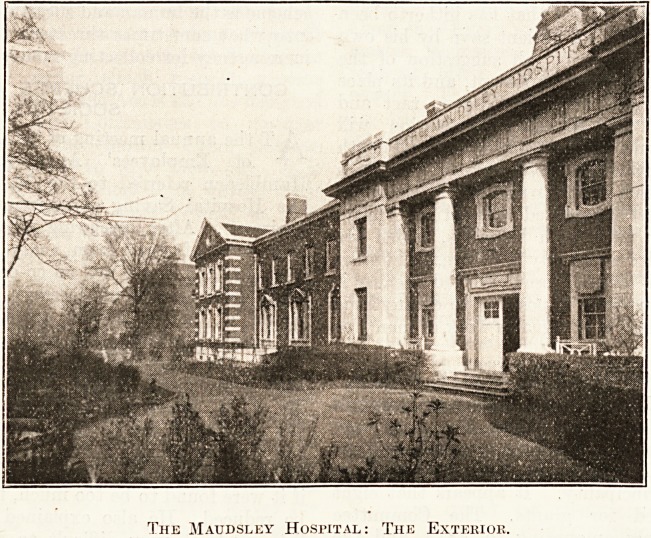


**Figure f2:**
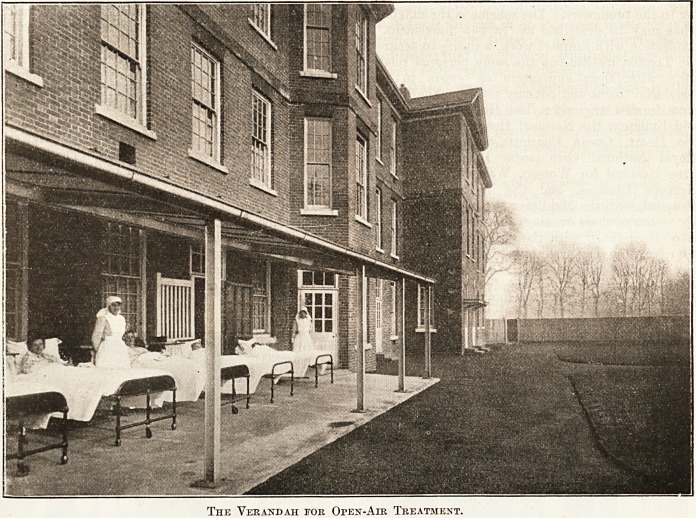


**Figure f3:**